# Aqueous *Ocimum gratissimum* extract induces cell apoptosis in human hepatocellular carcinoma cells

**DOI:** 10.7150/ijms.39436

**Published:** 2020-01-18

**Authors:** Chen-Cheng Huang, Jin-Ming Hwang, Jen-Hsiang Tsai, Jing Huei Chen, Ho Lin, Geng-Jhih Lin, Hsin-Ling Yang, Jer-Yuh Liu, Chiou-Ying Yang, Je-Chiuan Ye

**Affiliations:** 1 Institute of Molecular Biology College of Life Science, National Chung Hsing University, Taichung, Taiwan; 2 Division of Chest Medicine, Department of Internal Medicine, Taichung Hospital, Ministry of Health and Welfare, Taichung, Taiwan; 3 School of Applied Chemistry, Chung-Shan Medical University, Taichung 40201, Taiwan; 4 Basic Medical Science Education Center, College of Medicine and Health, Fooyin University, Kaohsiung, Taiwan; 5 Graduate Institute of Biomedical Sciences, China Medical University, Taichung, Taiwan; 6 Department of Life Science, National Chung Hsing University, Taichung, Taiwan; 7 Institute of Nutrition, College of Biopharmaceutical and Food Sciences, China Medical University, Taichung, Taiwan; 8 Center for Molecular Medicine, China Medical University Hospital, Taichung, Taiwan; 9 Department of Bachelor's Degree Program for Indigenous Peoples in Senior Health and Care Management, National Taitung University, Taitung, Taiwan; 10 Master Program in Biomedical Science, National Taitung University, Taitung, Taiwan

**Keywords:** * Ocimum gratissimum*, hepatocellular carcinoma, cell cycle arrest

## Abstract

Treatment of advanced hepatocellular carcinoma (HCC) has exhibited a poor overall survival rate of only six to ten months, and the urgency of the development of more effective novel agents is ever present. In this line of research, we aimed to investigate the effects and inhibitive mechanisms of aqueous *Ocimum gratissimum* leaf extract (OGE), the extract of *Ocimum gratissimum*, which is commonly used as a therapeutic herb for its numerous pharmacological properties, on malignant HCC cells. Our results showed that OGE decreased the cell viability of HCC SK-Hep1 and HA22T cells in a dose-dependent manner (from 400 to 800 µg/mL), while there is little effect on Chang liver cells. Moreover, cell-cycle analysis shows increased Sub-G1 cell count in SK-Hep1 and HA22T cells which is not observed in Chang liver cells. These findings raise suspicion that the OGE-induced cell death may be mediated through proteins that regulate cell cycle and apoptosis in SK-Hep1 and HA22T cells, and further experimentation revealed that OGE treatment resulted in a dose-dependent decrease in caspase 3 and PARP expressions and in CDK4and p-ERK1/2expressions. Moreover, animal tests also exhibited decreased HCC tumor growth by OGE treatment. We therefore suggest that the inhibition of cell viability and tumor growth induced by OGE may be correlated to the alteration of apoptosis-related proteins.

## Introduction

Hepatocellular carcinoma (HCC) is the fifth most common cancer type and the third leading cause of cancer-related death around the world 1-3, accounting for 75% to 85% of all primary liver cancer cases 4. The therapeutic approach for HCC depends on disease staging. Patients with early stage HCC are candidates for surgical resection, or radical therapy (cryosurgery, liver transplantation or local ablation via percutaneous ethanol injection (PEI) or radiofrequency ablation (RFA) with 5-year survival rates of 41-74% 5. For intermediate stage HCC, patients benefit from hepatic artery chemoembolization (TACE). However, patients with advanced HCC have very poor prognosis with an overall survival of only 6.5 to 10.7 months from sorafenib treatment 6-9. Therefore, the development of an effective novel agents with reduced toxicity is a priority to improve survival for advanced HCC.

*Ocimum gratissimum* is a commonly used herbal ingredient in traditional Chinese medicine and is widely distributed in tropical and warm temperate geo-locations. *O. gratissimum* aqueous extracts (OGE) with its many antioxidant components, has the potential to protect body organs from free radical damage and oxidative stress 10-19, and possesses many therapeutic functions, including anti-inflammation 20, analgesic and spasmolytic activities 21, antidiarrheal 22, antiviral 23 and anti-hyperglycemic activities 24, 25, and improves phagocytic function without affecting the humoral or cell-mediated immune system 26. In anti-cancer activity, the data have found that OGE can induce cell apoptosis in human lung adenocarcinoma A549 cells 27 and human osteosarcoma U2-OS and HOS cells 28. It is also able to modulate some cell cycle regulators (SKA2 and BUB1B) and apoptosis-related factors (PPP1R15A, SQSTM1, HSPA1B and DDIT4), which are reported to associate with drug resistance 29, 30. Moreover, in breast cancer, OGE inhibits cell chemotaxis and chemo-invasion *in vitro* and retards tumor growth and temporal progression *in vivo* 31. In prostate cancer 32, OGE inhibits cancer cell growth through reducing androgen receptor and survivin protein in a time dependent manner.

This study intends to investigate the cytotoxic and therapeutic effect of OGE on liver cancer cells, and the results showing that OGE can induce cell apoptosis and reduce tumor growth in HCC cells are discussed.

## Methods and Materials

### Materials

3-(4,5-dimethylthiazol-2-yl)-2,5-diphenyl-tetrazolium bromide (MTT), penicillin and streptomycin were purchased from Sigma (St. Louis, MO). Dulbecco's modified Eagle's medium (DMEM), fetal bovine serum (FBS) and trypsin-EDTA were purchased from Gibco BRL (Gaithersburg, MD). Antibodies against caspase 3, PARP, cyclin-dependent kinase 4 (CDK4), cyclin-dependent kinase 2 (CDK2), PFKFB3 and phospho-extracellular signal-regulated kinase1/2 (p-ERK1/2) were purchased from Cell Signaling Technologies (Beverly, MA). Antibodies against β-actin were obtained from Sigma. HRP-conjugated secondary antibodies against mouse IgG and rabbit IgG were purchased from Abcam Inc. (Cambridge, UK). The human hepatocellular carcinomaSK-Hep1 andHA22T cells, and one normal liver cell line (Chang-liver) were obtained from American Type Culture Collection (ATCC; Rockville, MD).The Mahlavu cells were gifted by Dr. Jaw-Ching Wu from the Institute of Clinical Medicine of the National Yang Ming University. The HA22T/VGH and SK-Hep-1 lines are malignant 33, 34.

### Preparation of *O. gratissimum* extract

Leaves of OG were harvested and washed with distilled water followed by homogenization with distilled water using a Polytron homogenizer. The homogenate was boiled for 1 h and then filtered through two layers of gauze. The filtrate was centrifuged at 20,000 g at 4^0^C for15 min to remove insoluble pellets and the supernatant (OGE) was thereafter collected, lyophilized and stored at -70^0^C until use.

### Cell Culture and Experimental Treatments

All cells were cultured in DMEM or RPMI 1640and supplemented with 10% FBS and 100 μg/mL penicillin/streptomycin at 37^0^C in a humidified atmosphere containing 5% CO_2_. The HCC cells were maintained in 100 μM non-essential amino acid, 2 mM glutamate. Cells were seeded in culture plates and grown to approximately 80% confluence. Cells (4 x 10^4^cells/mL) were then transported to experiment culture plates and maintained at 37^0^C in a humidified atmosphere containing 5% CO_2_.After 48 h, the cells were treated with OGE at indicated concentrations for the indicated hours and then collected for the following analyses.

### MTT Assay for Cell Viability

Cell viability was determined by MTT assay after treatment of the cells with 0, 100, 200,400, 600and 800 μg/mL OGE for 24, 48 and 72 h. After the treatments, medium was removed, and cells were incubated with MTT(3-(4,5-Dimethylthiazol-2-yl)-2,5-diphenyltetrazolium bromide) (0.5 mg/mL) at 37^0^C for 2 h. The viable cell number was directly proportional to the production of formazan, which was dissolved in isopropanol and determined by measuring the absorbance at 570nm using a microplate reader (Spectra MAX 360 pc, Molecular Devices, Sunnyvale, CA).

### Cell Cycle Analysis by Flow Cytometry

The cell cycle was analyzed by flow cytometry after treatment of the cells with 0, 400, 600 and 800 μg/mL OGE for 48 h. All of the cells, cells in the suspension and adherent cells, were collected, washed, and suspended in cold PBS. Cells were then fixed in chilled 75% methanol and stained with propidium iodide (PI). Analysis was performed in the FACSCalibur flow cytometer running CellQuest (Becton Dickinson, San Jose, CA).

### Western Blotting Analysis

Cells were washed with PBS and lysed with lysis buffer (50mM Tris-HCl, pH 7.5, 150mM NaCl, 1% Nonidet P-40, 1mM phenylmethylsulfonyl fluoride, 1mM sodium fluoride, and 10 μg/mL aprotinin and leupeptin) after treatment of the cells with0, 400, 600, 800 μg/mL OGE for 24 h. The lysates were put on ice for 30 min and then centrifuged at 20,000 g for 15 min. The supernatants were collected and measured for protein concentration using the Bradford method. Crude proteins (30 μg per lane) were subjected to a 12.5% SDS-polyacrylamide gel, and then transferred onto a nitrocellulose membrane (Millipore, Bedford, MA). The blotted membrane was then blocked with 5% w/v skimmed milk in PBS, and then incubated for 2 h with 1/1000 dilution of antibodies against human Caspase 3, PARP, p-ERK1/2, CDK4, CDK2, PFKFB3, and β-actin. β-Actin protein was used as an internal control. Antigen-antibody complex was detected using 1/2000 dilution of peroxidase-conjugated secondary antibodies and displayed using ECL chemiluminescence reagent (Millipore, Bedford, MA).

### Bioenergetic assay

Analysis of oxygen consumption rate (OCR) and extracellular acidification rate (ECAR) were performed using a Seahorse XFe Flux Analyzer (Seahorse Bioscience). SK-Hep1 cells were seeded into XF 24-well cell culture microplates with serum-free DMEM in Extracellular Flux (XF) media (non-buffered RPMI 1640 containing 25 mM glucose, 2 mM L-glutamine and 1 mM sodium pyruvate). Cells were then transferred to a CO_2_ free incubator, maintained at37^0^C in a humidified atmosphere overnight. The SK-Hep1 cells were then treated with 0, 200, 400, 600 μg/mL OGE immediately before the assay. Following instrument calibration, the OGE treated cells were transferred to the XFe Flux Analyzer to record OCR and ECAR in 200, 400, 600 minutes.

### Tumorigenicity Assay in Nude Mice

Female BALB/c nude mice, 4-6 weeks of age, are purchased from National Health Research Institute (Taipei, Taiwan, ROC), housed in a dedicated nude mouse facility with microisolator caging. The Mahlavu cells are detached by trypsinisation 48 h later, and then washed in triplicate in serum-free DMEM. 1 × 10^7^ cells in 20 μl volume is injected into the liver organ of mice by use of a 0.3 ml insulin syringe with 29-gauge needle. Two weeks after injection, the mice are separated into control (water)-and60mg/kg OGE-oral-injected groups. There are 2-3 mice in each group. One month after treatment, the tumors and organs were harvested from the sacrificed mice and weighed.

### Statistical Analysis

Data were expressed as mean ± SEM and analyzed using analysis of variance (ANOVA). Student's t-test was used in two-group comparisons. P <0.05 was considered to be statistically significant.

## Results

### OGE Inhibits cell viability of HCC cells

**Figure 1:**
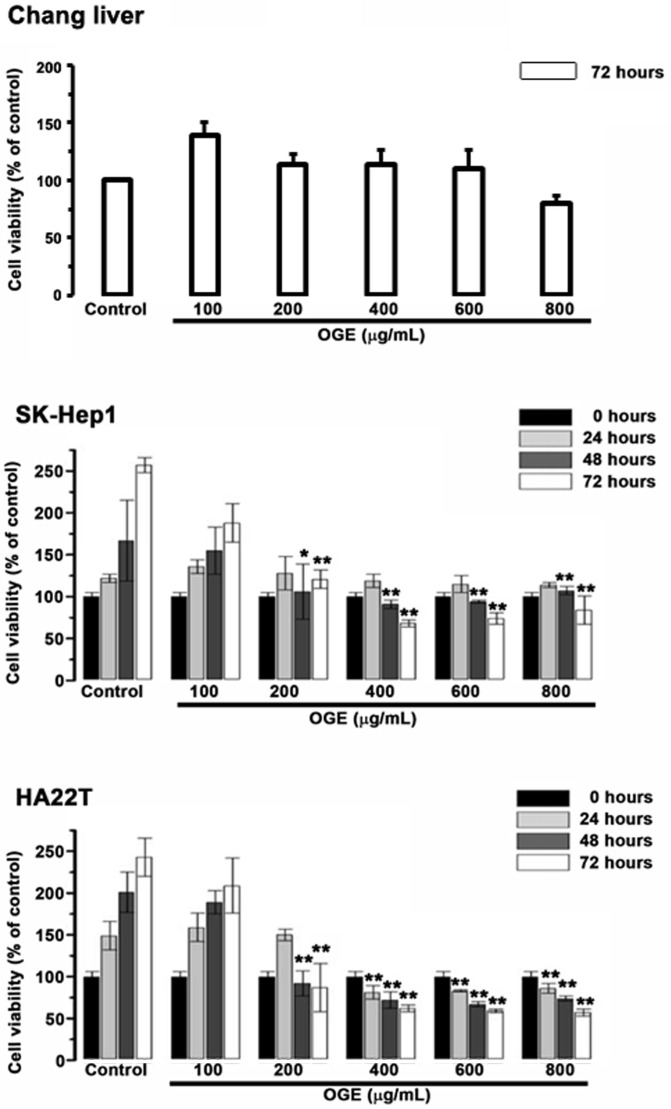
Effects of OGE on the viability of the Chang liver, SK-Hep1 and HA22T cells. Cell growth was determined 24, 48, and 72 h after OGE treatment using the MTT assay as described in the Materials and Methods. Absorbance values obtained from the cells on hour 0 of treatment on subculture were taken as 100%. The data indicate mean ± SE (*n*=3 in each group). **p < 0.01,*p < 0.05 as compared with control 0 μg/mL OGE group.

To examine the cytotoxic effects of OGE on the HCC cells and the Chang liver cells, they were treated with serial concentrations of OGE (0,100, 200, 400, 600, 800 μg/mL) for0, 24, 48 and 72 h and subsequently analyzed for cell viability by using MTT assays (Figure 1). In SK-Hep1 and HA22T cells, cell viability significantly decreased compared to control when OGE concentration was above 200 μg/mL at 48 and 72 h (Figure 1). There was no statistically significant change of cell viability observed on the Chang liver cells below 800 μg/mL at 72 h.

### OGE induces morphological alteration in HCC cells

**Figure 2:**
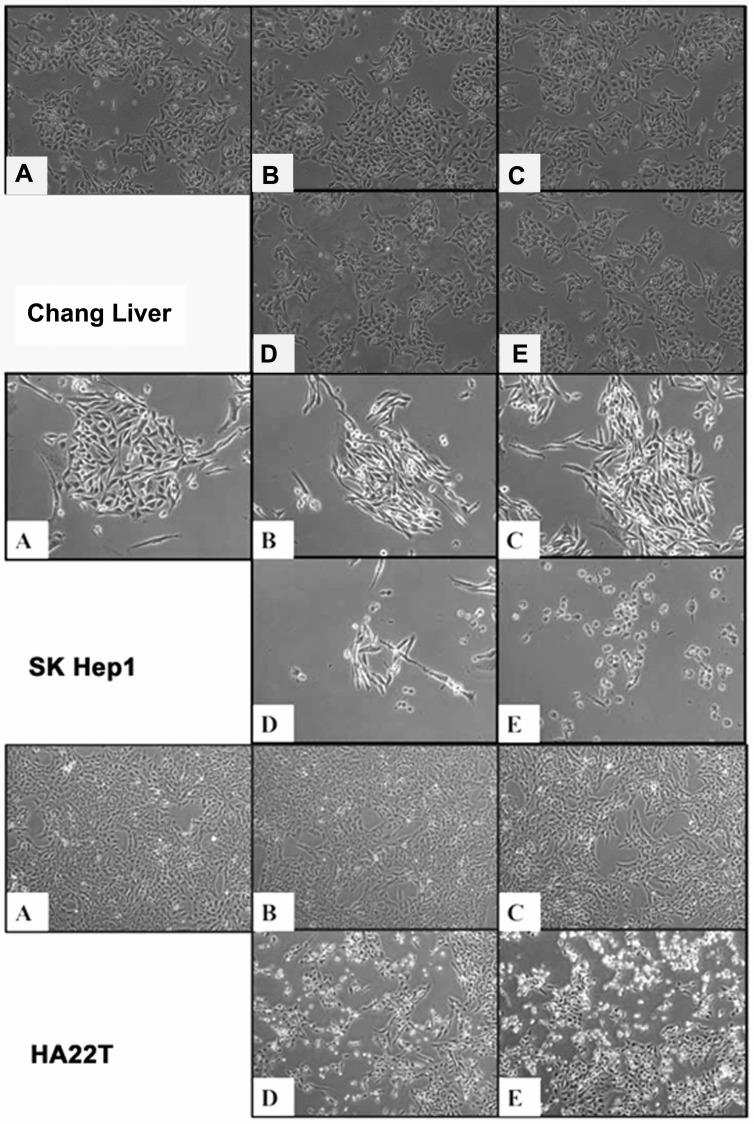
Morphological change in Chang liver, SK-Hep1 and HA22T cells by (A) 0, (B) 200, (C) 400, (D) 600, (E) 800 μg/mL OGE treatment at 48 h at 400 x magnification.

To observe the cell morphology, the HCC cells treated with different concentrations (0, 200, 400, 600, 800 μg/mL) of OGE were examined at 48 h using Nikon Digital Sight DS-L1(Figure 2). Cell Shrinkage and detachment were observed on the SK-Hep1 and HA22T cells in a dose-dependent manner. No morphology change was detected in OGE-treated Chang liver cells.

### OGE changes cell cycle in HCC cells

**Figure 3:**
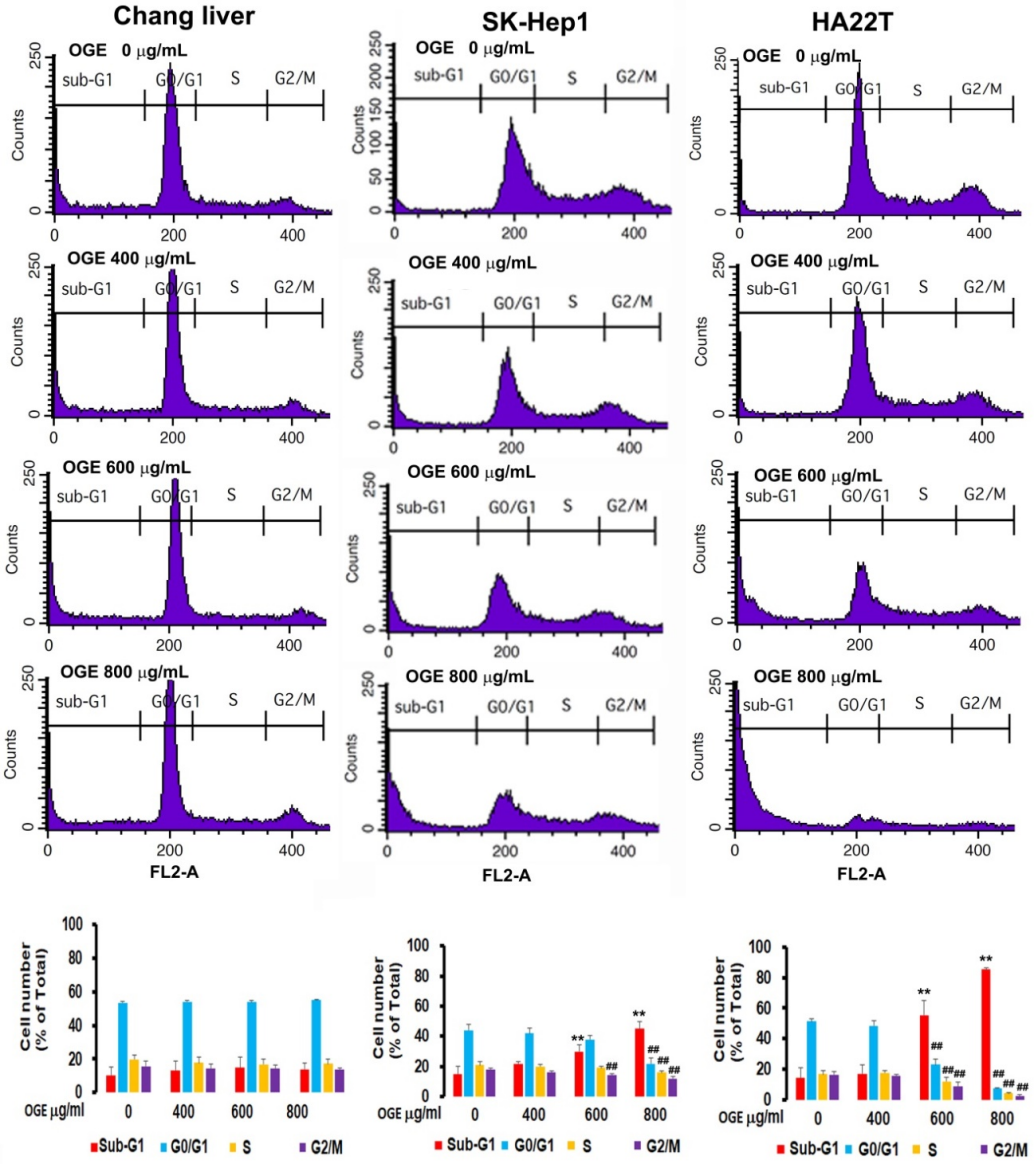
Cell cycle distribution by flow cytometry analysis in Chang liver, SK-Hep1and HA22Tcells after exposure to OGE. The cells were treated with 0, 400, 600, and 800 μg/mL OGE for 48 h. Results are plotted as the percentage of remaining cells in each cell cycle phase. Data is presented as mean ± SE of three independent experiments. The data indicate mean ± SE (*n*=3 in each group). **p < 0.01up-regulation as compared with control group; ^##^p < 0.01down-regulation as compared with control group.

To investigate the effects of OGE on cell cycle, cells treated with different concentration of OGE (0,400, 600, 800 μg/mL) for 48 h were collected for cell cycle analysis using BD FACSCalibur Flow Cytometer. The results showed that the cell number in sub-G1 phase in SK-Hep1 cells was significantly increased by 47%, 92%, and 178% compared to 0 dose control group in 400, 600 and 800 μg/mL of OGE respectively (Figure 3), and in HA22Tcells was significantly increased by 18%, 283%, and 489% compared to 0 dose control group in 400, 600 and 800 μg/mL of OGE respectively, while the cell number in the rest of the cell cycle phases were relatively decreased, indicating a certain level of cell death. Whereas, no significant change was observed in Chang liver cells.

### OGE changes the expressions of the cell-apoptosis- and cell-cycle-related checkpoint proteins in HCC cells

**Figure 4:**
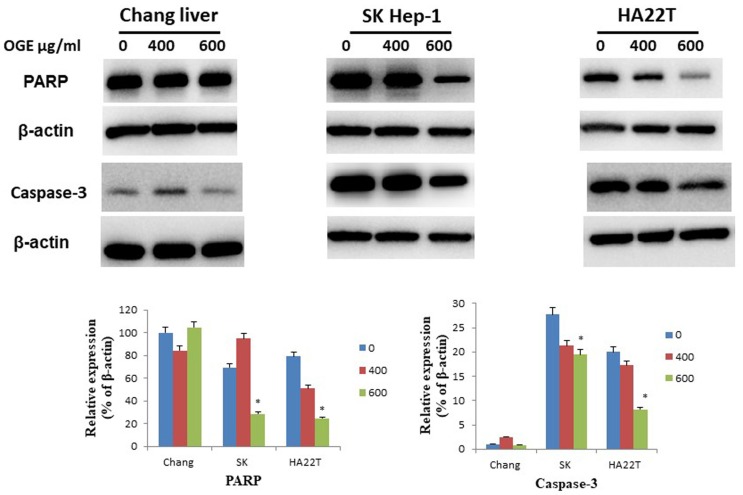
Expression of caspase 3and PARP in Chang liver, SK-Hep1 and HA22T cells measured by immunoblotting analysis. Cells were treated with 0, 400, and 600 μg/mL OGE for 24 h. Immunoblotting analysis were performed as described in the Materials and Methods. Control cells (treated with 0 μg/mL OGE) were treated with vehicle alone. The data indicate mean ± SE (n=3 in each group). *p < 0.05 down-regulation as compared with control group.

To determine whether OGE could affect cell apoptosis-related proteins, we examined the expressions of caspase 3 and PARP by immunoblotting in HCC cells after treatment with different concentrations (0, 400, and 600 μg/mL) of OGE for 24 h. In SK-Hep1 and HA22T cells, PARP and caspase 3 levels decreased (Figure 4) in the treatment of 600 μg/mL of OGE. Whereas, no significant change was observed in Chang liver cells in the above concentrations.

**Figure 5:**
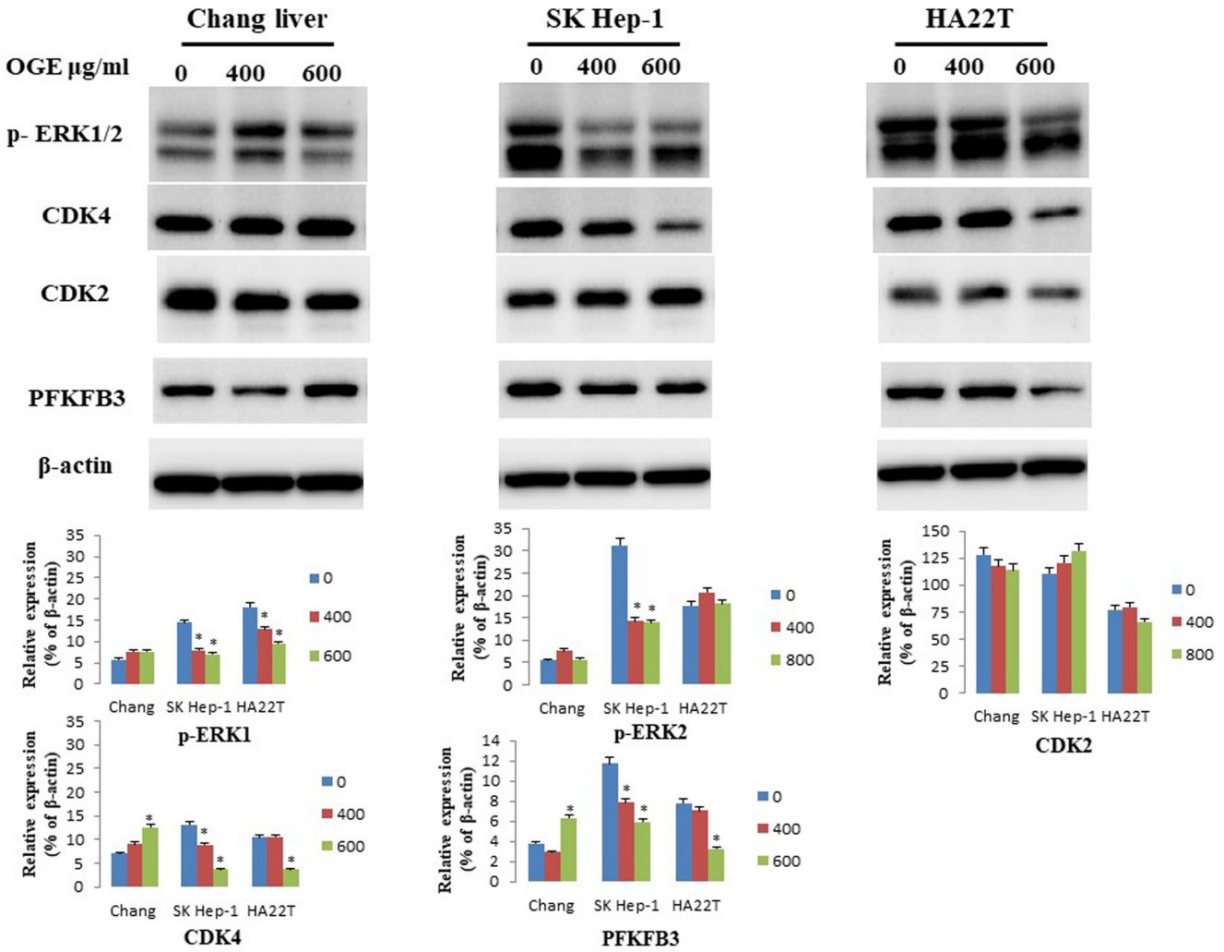
Expression of CDK4, CDK2,p-ERK1/2 and PFKFB3in Chang liver, SK-Hep1 and HA22T cells measured by immunoblotting analysis. Cells were treated with 0, 400, and 600 μg/mL OGE for 24 h. Immunoblotting analysis were performed as described in the Materials and Methods. Control cells (treated with 0 μg/mL OGE) were treated with vehicle alone. The data indicate mean ± SE (*n*=3 in each group). *p < 0.05down-regulation as compared with control group.

To determine whether OGE could affect cell cycle related checkpoint proteins, we further examined the expressions of CDK4, CDK2, p-ERK1/2, and PFKFB3 by immunoblotting in HCC cells after treatment with different concentrations (0, 400, and 600 μg/mL) of OGE for 24 h. In SK-Hep1 and HA22T cells, the protein levels of CDK4, p-ERK1/2, and PFKFB3decreased (Figure 5). No change was observed in CDK2 levels in either cells, and no significant change in all checkpoint proteins was observed in Chang liver cells.

### OGE significantly decreases ECAR of glycolysis in HCC cells

It is known that malignant cells such as HCC cells prefer to consume energy from the process of aerobic glycolysis but not from mitochondria function 35. CDK4 has been identified as oncogenic when alongside other genes, such as ERK, c-Myc, and cyclin D1, and participates in the glycolytic process 36, 37. CDK4 itself was found to promote glycolysis 38 and potentially acts as a positive clinical prognostic marker for malignant phenotypes of HCC 39. In the above data, OGE has been observed to inhibit CDK4, and we hypothesized that OGE can affect the glycolytic process through this pathway and not the mitochondria pathway.

**Figure 6:**
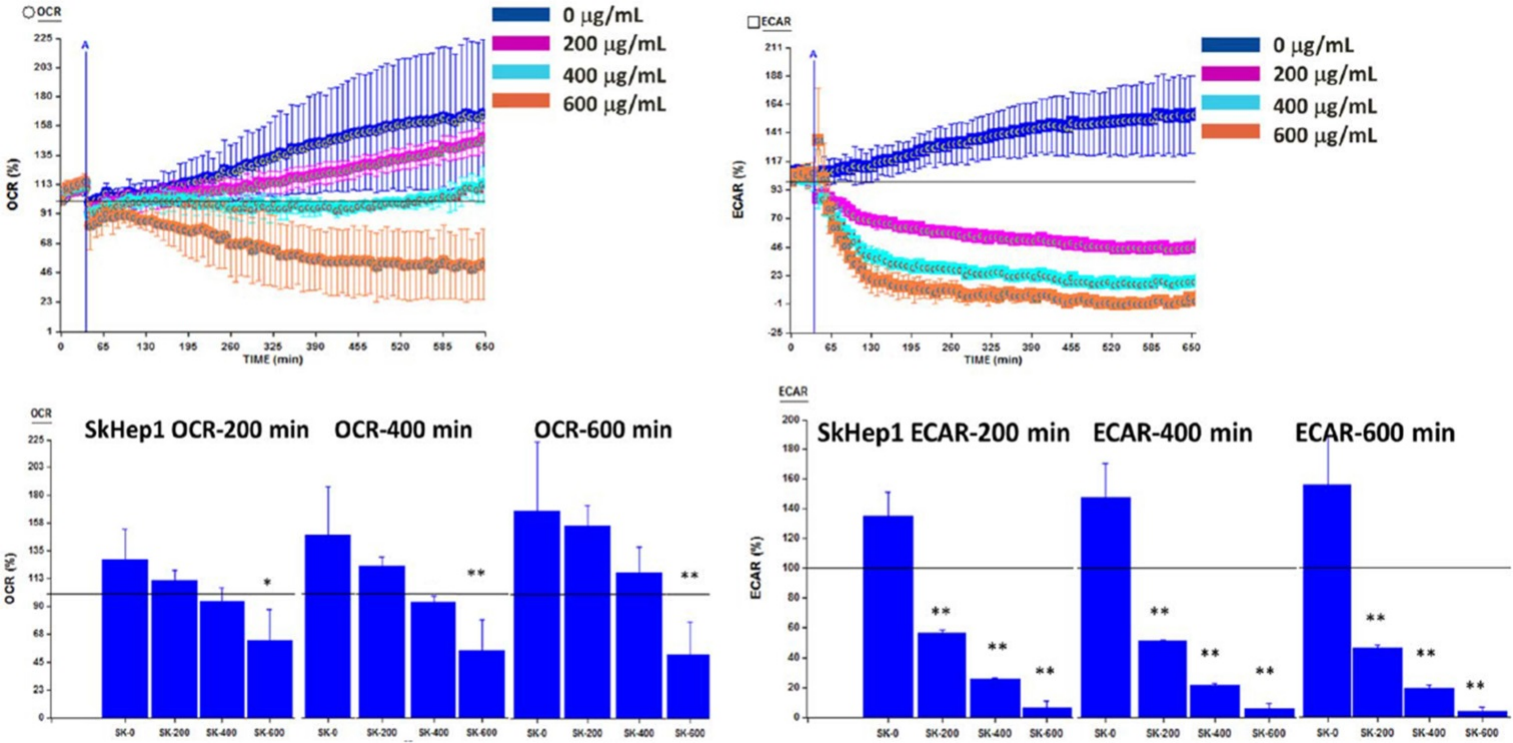
Oxygen consumption rate (OCR) and Extracellular acidification rate (ECAR) in SK-Hep1 cells measured by Bioenergetic assay.SK-Hep1 cells were treated with 0, 200, 400, and 600 μg/mL OGE for 600 mins. Bioenergetic assay were performed as described in the Materials and Methods. Control cells (treated with 0 μg/mL OGE) were treated with vehicle alone. The data indicate mean ± SE (n=3 in each group). **p < 0.01,*p < 0.05 as compared with control 0 μg/mL OGE group.

To investigate whether OGE reduces cell viability through inhibiting glycolysis or mitochondrial function, ECAR and OCR were analyzed using a Seahorse XFe Flux AnalyzeronSK-Hep1 cells at OGE concentrations of 0, 200, 400 and 600 μg/mL at the indicated timeframes. ECAR was significantly decreased by 88%, 116%, and 129% compared to control group at 200 minutes, by 96%, 131%, and 140%at 400 minutes, and by 116%, 142%, and 151% respectively at 600 minutes (Figure 6). Whereas, OCR was significantly decreased by 65%, 90% and 93% compared to control group only in 600 μg/mL of OGE for 200, 400 and 600 minutes respectively. This suggests the effect of OGE on the glycolytic process over the mitochondrial function.

### OGE significantly decreases tumor growth

**Figure 7:**
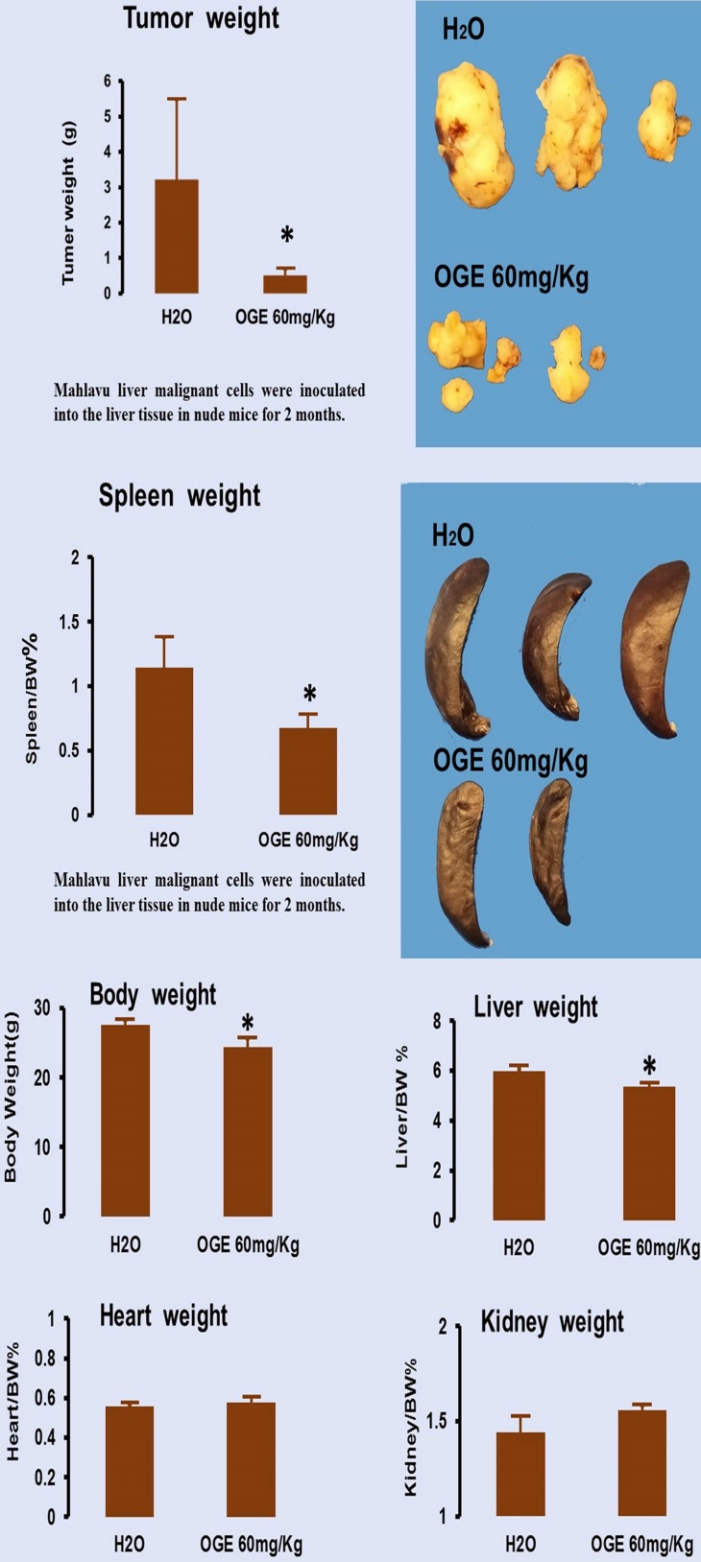
Tumor growth in nude mice after xenografts of hepatocellular carcinoma Mahlavu cells. Cancer removed from the mice were weighed (upper panel) and other organs removed from the same mice were weigh (middle and bottom panels). **p< 0.01 compared with control group.

To evaluate the therapeutic effects of OGE, nude mice bearing Mahlavu cell xenografts were orally injected with 60 mg/kg OGE daily for one month. As shown in Figure 7, compared with the tumor size in the control group, OGE-treated mice had smaller tumor sizes (Figure 7, upper panel). The mean weight of tumors of the control group was 3.1±2.2 g and 0.5±0.2 g for the OGE group, demonstrating that OGE significantly suppressed tumor weight compared to the control group. The spleen weight was decreased (Figure 7, middle panel) and the other organs showed no change in weight (figure 7, bottom panel).

## Discussion

Previously, OGE was reported to have cytotoxic effect on several human solid tumor cell lines including human lung, breast, cervical, bone and prostate cancers 40. This study showed that OGE also had cytotoxic effect on HCC SK-Hep1and HA22T cells with morphological alterations and condensation changes and in a time- and dose-dependent manner. These findings are consistent with the results of cell-cycle and Western blot analyses, and those of our other studies 27, 28 which show that OGE significantly and dose-dependently modulate the expression of apoptosis-related proteins of cancer cells, indicating the activation of cell apoptosis. However, cytotoxic activity was absent on Chang liver cells, suggesting the selectivity of OGE on specific properties of cancer cells.

Numerous human cancers, including liver cancer, are known to feature aberrant extracellular signal-regulated kinase (ERK) expressions 41, and ERK is known alongside numerous protein kinases, such as c-Jun N-terminal kinase (JNK), andp38 MAP kinase (p38), to make up the core of mitogen-activated protein (MAP) kinase pathway cascades. In HCC tissues, increased expression of ERK was correlated with poor disease-specific overall survival 42, which maybe a contributing factor to the malignancy and high frequency of metastasis of liver cancer. In this study we found decreased p-ERK1/2 levels in the HCC cells after OGE treatment, suggesting the influence of OGE on survival signaling of liver cancer cells with high ERK activity through an unknown mechanism.

The mechanism suspected to connect high ERK activity to survival signaling is aerobic glycolysis, which is promoted when ERK1/2 phosphorylates pyruvate kinase muscle isozyme (PKM1/M2) that translocate into the nucleus and upregulates the expressions of c-Myc, cyclin D1 and CDK4, which also promote cell viability 44. The existence of a link between aerobic glycolysis and tumorigenesis has been known for several decades as the “Warburg effect” 45. Cancer cells have a unique metabolism of performing lactic acid fermentation in the presence of oxygen. Since higher glycolytic rate in tumor cells can promote resistance to chemotherapeutics 46, 47, the interruption or even disruption of tumor glycolysis can impact tumor growth by energy depletion as well as sensitization to therapeutics. Moreover, CDK4 has been identified as oncogenic when alongside other genes and participates in the glycolytic process 48, and CDK4 itself was found to promote glycolysis 38 and potentially acts as a positive clinical prognostic marker for malignant phenotypes of HCC 39.

In the bioenergetic assay on SK-Hep1 cells (Figure 6), OGE above 200 μg/mL significantly decreased ECAR of malignant HCC cells, whereas OCR was significantly decreased only above 600 μg/mL. In Western blot, CDK4 level decreased with increasing concentration of OGE treatment in the HCC cells. Further evidence of change in glycolysis was found in the decrease of 6-phosphofructo-2-kinase/fructose-2,6-biphosphatase 3 (PFKFB3) in HCC cells (data not shown), which is an allosteric activator of 6-phosphofructokinase-1 (PFK-1), a glycolysis stimulator. These findings suggest that the decreased cell viability of HCC cells by OGE treatment may be correlated with the inhibition of aerobic glycolysis. Similarly, a paper has shown that genistein, a natural isoflavone, induced HCC cell apoptosis by directly suppressing aerobic glycolysis 49. However, since tumor weight from mice injected with Mahlavu cells significantly decreased after OGE treatment (Figure 7), we suggest that OGE treatment may cause tumor growth decrease by modulation of ERK signaling pathway and aerobic glycolysis and increasing cell apoptosis.
